# COVID-19 Rehab Fright Management

**DOI:** 10.12669/pjms.37.1.3187

**Published:** 2021

**Authors:** Nazia Mumtaz, Ghulam Saqulain, Nadir Mumtaz

**Affiliations:** 1 Dr. Nazia Mumtaz, PhD (Rehabilitation Sciences) Head of Department of Speech Language Pathology, Faculty of Rehab & Allied Health Sciences, Riphah International University, Islamabad; 2 Dr. Ghulam Saqulain, F.C.P.S (Otorhinolaryngology) Head of Department of Otolaryngology Department of ENT, Capital Hospital, Islamabad; 3 Mr. Nadir Mumtaz, LLB Former DG Research, FBR Islamabad

**Keywords:** COVID-19, Otolaryngologists, SARS-CoV-2, Speech Language Pathologists, Telehealth, Tele-rehabilitation

## Abstract

Being diagnosed as positive for SARS-2 CoV RNA on PCR generates anxiety and stress as well as depression due to the prospects of being isolated. With genetically varied forms of virus on the rise the widely adopted manner to protect oneself is social distancing. This is frightening for rehabilitation professionals who at times are exposed at close quarters to the patients as rehabilitation is essential. Data in this backdrop is lacking, as this is a recent and ongoing pandemic. As such the current study was conducted with an attempt to review and highlight the causes of fright among rehabilitation professionals and possible management options in the wake of current pandemic of COVID-19 in the perspective of Pakistan. For this purpose literature was searched from major electronic databases including PubMed, Google, Google Scholar and Web-of-science, with keywords “Covid-19, mental health, telehealth, telemedicine, tele-rehabilitation and combination of words”. Eighty English, full text articles were studied out of which 36 were used for the literature review. With this literature review we conclude that COVID-19 has resulted in fear of contracting and transmitting this disease among health professionals and can be reduced and managed by provision of tele-rehabilitation and telehealth facilities. Patients emerging from prolonged mechanical ventilations require extensive rehabilitation to restore routine body functions. The role of the otolaryngologist and speech language pathologist (SLP) is formal and direct to ensure appropriate and timely long term intervention and rehabilitation to ensure that these individuals re-enter mainstream activities.

## INTRODUCTION

The mere fact that one has tested positive for COVID-19 leads to anxiety and stress whereas remaining isolated in order to minimize and avoid exposure and reducing transmission with its accompanying loneliness leads to depression^1^ and a sedentary lifestyle which is a classic ingredient for a non-healthy lifestyle may lead to health issues including overall increased psychological burden^2^, cardio metabolic diseases as well resulting in increased morbidity and mortality.^3^ Social isolation and lack of human interface, which cannot be compensated by video interaction, is likely to result in lowered human productivity in the areas of manufacturing, research and innovation as the primary urge to reach out and test one’s own limits is no longer there. Webinars being mostly one-way dialogues or monologues are a temporary phase. Podcasts cannot be a substitute for international conferences, workshops and seminars and are being bandied about by their protagonists with declining appeal and utility. Eventually stringent manner of disease mitigation will be tapered down. There is yet no evidence-based postulate to suggest that COVID-19 is prevalent amongst different races, DNA strand, or gender and individuals with intellectual disabilities except an understanding that the aged population suffering from dementia is more susceptible^4^, hence people with dementia in the community demand attention.^5^ Higher fatalities have been noted in individuals with autism and intellectual disabilities.^6^ Co-morbidity may also play a role in fatalities attributable to COVID-19. Perhaps genetically varied forms of virus are mutating^7^, and rapidly replicating as we speak thereby discarding the notion that certain individuals have an inherent resistance or the so-called “herd immunity”. Nearly every individual contracts influenza. That may be one factor why the discovery of the vaccine by the world’s pharmaceutical conglomerate with an assured and captive audience is still elusive. The medically scaring scenario is that health care institutions resources are stretched. Conservative estimates as far back as 2008 placed the number of patients needing extended mechanical ventilation at 600,000^8^, however this pandemic of 2020 challenges all previous surmises

According to Koh GCH et al, unfortunately, the only way to protect ourselves are measures to prevent infection and enforce social distancing^9^, engendering fright for rehabilitation professionals expending considerable time with patients. Rehabilitation is essential and has benefits in acute as well as recovery stages of COVID-19 to enhance respiratory function, endurance to exercise, psychological help and self-care 10 but there are fears for rehab professionals as well which need to be managed. Hence research is vital to establish mandatory rehabilitation programs.^10^

**Figure 1:**
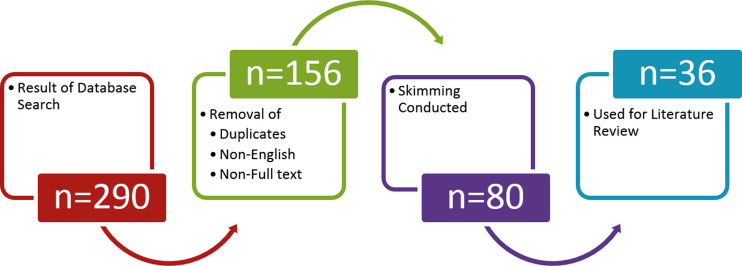
Schematic representation of literature search.

With lack of data related to this, the current study was conducted with an attempt to review and highlight the causes of fright among rehabilitation professionals and possible management options in the wake of current pandemic of COVID-19 especially with the perspective of Pakistan. For this purpose literature was searched from major electronic databases including PubMed, Google, and Web-of-science, with keywords “Covid-19, mental health, telehealth, telemedicine and combination of words”, with no publication date limitations. Our search (Fig.1), revealed around 290 articles and news, from which non-English and duplicates and in those where full texts were not available were removed leaving behind 156 downloaded articles. This was followed by skimming for relevant titles which identified 80 articles, out of which 36 were used for the manuscript’s literature review.

## DISCUSSION

In Pakistan we are encountering paranoia as a direct fallout of the viral syndrome severe acute respiratory disease coronavirus 2 (SARS-CoV-2) termed COVID-19. The novel aspect of this viral syndrome is that it manifested in the developed and affluent countries and societies and financial centers almost simultaneously. The positive outcome was that these countries had the financial reserves and social responsibility to a mostly self-imposed lockdown, which spread throughout, even in the developing world.

Through a questionable use of security dragnet and the power of the social media the un-informed, illiterate, ignorant and non-healthcare individuals complied even at risk to day to day livelihood with losses of income in the developing countries being estimated to be above $220 billion.^11^ Also in high and middle income states children were luckily less affected, however low and middle-income countries are fearful of exposure of already vulnerable children due to prevalence of risk factors of pneumonia.^12^

Though how efficacious the face mask is needs to be evaluated, however the mask seems to be the commonly available defense for containment and mass masking has been recommended.^13^ Quarantine, isolation, social distancing as well as containment of contagion in community is required to halt the spread of disease^14^ all causing an environment of fear for the public as well as rehabilitation specialists alike. Recent research in the USA indicates that flattening of the fatality and infection curve may be attributable to wearing face masks and social isolation, hence, universal use of face mask has been recommended with significant impact on outbreak.^15^ In Pakistan the percentage of working women is less and women remain restricted to the home where the impact of social isolation on this gender may be significantly greater and mental issues may become prevalent.

Researchers caution that the spread of COVID-19 may be slowed as social isolation and face covering prevent both airborne transmission by blocking atomization and inhalation of virus-bearing aerosols contact transmission with the research implication that spread of COVID-19 will slow till vaccines to stop the epidemic come on line.^14^ Yet transmission will not stop thereby implicitly warning that spurts and spikes will continue to take place in Pakistan and in other regional countries. Pakistan’s healthcare system has small number of 1650 ventilators, with a health spending of 0.75% of GDP, the requirements might not be met if there is exponential increase in COVID-19 cases.^16^ Pakistan’s preventive, acute medical care infrastructure and rehabilitative health care needs to be drastically revamped and funding significantly enhanced commensurate to any surge in COVID-19 patients.

The constant threat of COVID-19 may lead to an upheaval in rehabilitation care. The long term effects of COVID-19 on dysphagia are interlinked with present day evaluation and treatment and non-discriminately applicable to any future healthcare crisis accompanied by relatable constraints.^17^

Anxiety and fear of touching a possibly infected surface, or hearing anyone coughing and sneezing in close proximity can generate temporary mental health fears in normal individuals but can significantly impact those receiving rehabilitation further compounded by the visible impact of observing healthcare workers wearing PPE’s.^18^ Some form of refreshing therapeutic activity is required.^18^

In Pakistan frontline workers in rehab include otolaryngologists, speech and language pathologists, occupational therapists and physiotherapists are exposed to COVID-19 patients once they are being weaned from critical life care surroundings or post intubation. Rehabilitation professionals indulge in an integrated, dynamic and constructive inter disciplinary manner and are brazenly exposed to the anatomical and physiological inner core of the virus while in the clinical evaluation and treatment of the oropharynx, nasopharynx, larynx, and upper and lower respiratory airways. Physical proximity to COVID-19 patients is disconcerting and dangerous yet adopting maximum protection can mitigate risks encountered by otolaryngologists, medical and rehab professionals in acute care settings.^19^ Patients emerging from post-extubation and mechanical ventilation in critical care environment are the prime candidates for such clinical and close proximity engagement. The intensive care physician determines the safe limits of swallowing of fluid and semi solids which is a delicate balance between the degrees of airway risk in acute care settings. The speech language pathologist clinically manages the swallowing protocol. The COVID-19 patient care is a slowly developing protocol and to a certain degree the primary healthcare physicians, otolaryngologists and rehabilitation professionals like SLP’s are a factor in risk stratification of airway vulnerability with noninvasive imaging and noninvasive metrics.^17^ In a local study by Urooj U et al. reported worries of doctors at all levels of hospital functioning especially fear of their families getting infected (79.7%), and themselves acquiring infection or being vectors (28.8%) in wake of PPE shortages, rapid disease spread 63%), complications of COVID-19 (60.3%) and missing diagnosis ( 27.9%).^20^

With clinical treatment of COVID-19 patients is at a nascent stage evaluation criteria encompasses swallowing characteristics as laryngeal structure and dynamics, lingual deformation during swallowing, airway compromise during swallowing, and efficiency of swallowing frequency and physiology in order to provide and maintain safe nutritional status of such patients as management protocols. On the rehabilitation side SLP’s perform a number of procedures, which initiate cough reflex and consequential aerosol generation. Obviously professionals are alarmed when conducting procedures for difficulty in speech and swallowing. The DRS COVID-19 Taskforce has summarized the AGP’s which include basic procedures like oral care and cough reflex testing which transmits infection.^21^ Such procedures entail aerosol droplets landing on the mucosal surfaces of eyes, nose and mouth causing infection^22^, especially tracheostomy.^23^

Stroke management has become a challenge making clearly defined treatment and rehabilitation plans including telemedicine as well as virtual consultations the need of the time.^24^ Elaborate precautions are mandated for healthcare workers encountering suspected cases with viral infections or positive cases of COVID-19 in which case N95 masks or special powered air purifying respirators (PAPR), shields/ googles/ gloves and gowns mandatory when performing procedures involving the larynx.^21^ The efficacy of alternatively making available telemedicine multipurpose systems for healthcare through digital platform for patients and healthcare worker are at a nascent stage.^25^ Rehab post COVID-19 demands multidisciplinary team service and preparation to deal with the aged in need of rehabilitation due to isolation, social distancing, and restriction of movement^26^ with deconditioning and enabling rehabilitation for those quarantined. Simultaneously health care provider needs to be made aware that excess application of emollient and steroids local application, hermetically sealing of masks and decontamination of equipment is not recommended 9. Rehabilitative services may be required in those COVID-19 cases with existing comorbidities and productive cough with fear of infection spread.^27^

Patients of COVID-19 with depression abound and require alternate digitally based online therapies^28^ to alley the fear of COVID-19 infection and reassure patients. An article by Shah K et al. highlights that previous outbreaks of such contagious disease impacted healthcare workers as well as patients beset with cognition deficits with infiltration of anxiety, emotional distress, fear and even evidence of post-traumatic stress .Never was the need for counseling been more.^29^ Shanafelt T et al. reported eight anxiety concerns of healthcare professionals ranging from availability of PPE’s, exposure to infection at work and transmission to relatives non-access of rapid COVID-19 tests, change in work area with deficient knowledge and competence for that and access limitations to contemporary knowledge. Such apprehensions need to be actively addressed to satisfy and establish a care framework for healthcare professionals as well as dedicated medical service availability for them.^30^ Better provision of psychological help and services play a vital role as well as balanced and comprehensive plans to cater to future stresses.^31^ The incidence of strokes have increased as has dysphagia, with options of telehealth during this pandemic.^32^ In Pakistan the realization of telehealth is there since 2005 with establishment of satellite based telemedicine system in three major cities^33^ however further progress was not seen. There is now an understanding emerging that standardized telehealth rehabilitation through speech language professionals may serve to reduce post hospital discharge financial burden on the patient as well on the health care system but no serious thought has been made by the official quarters. Telehealth has its limitations in Pakistan on account of awareness and understanding issues and as time clinical rehabilitation pathways become increasingly available it would reduce the load on hospitals and prevent re-hospitalization. Zahid Z et al have predicted bright future for tele-rehabilitation in Pakistan, with the condition that non-governmental organizations and policy makers take up the issue or launching of a nationwide program.^34^ This would cater to the essential requirement of a side-by-side digitally accessible data receptacle maintained by the federal and provincial authorities for standardization of treatment techniques and stimulate COVID-19 related research, and thus help in playing down the fear of COVID-19. However with average knowledge of telemedicine in the health professionals in Pakistan, workshops and conferences are the need of the hour.^35^ In one study by Stephens W, the authors reported readmission rate of 6.2% compared to control of 18.1% using video telehealth pulmonary rehabilitation.^36^ Rehab professionals like SLPs, OTs and PTs are at increased risk due to close proximity and direct exposure to patients.^9^

One keeps hearing about rise or flattening of curves. The burden of rehabilitation of seriously affected COVID-19 patients will linger on for a considerable period and the policy makers at the federal and provincial levels need to cater to this. The surge of COVID-19 has commenced and may not be over soon as the population of Pakistan is many times more than the most affected European countries. Pakistan would not want to be categorized as a COVID-19 region. To compound matters Pakistan’s health authorities severely restricted hospitals outpatient inflow ostensibly to divert the existing heath care infrastructure to manage critical care patients.

## CONCLUSIONS

New frontiers of science have to be breached. Eventually the world will learn to live with COVID-19 as with other contagious diseases, which afflict individuals with compromised immune systems. It is imperative that the pharmaceutical industry of Pakistan, the federal and provincial health authorities, Pakistan’s speech and language pathologist professionals registered association, otolaryngologists’ forum and health regulatory bodies develop and establish initiatives for COVID-19 focusing on everyday rehabilitative techniques and routine value added care. Traditional skepticism and reluctance need to be quelled when innovation is the clarion call of the day in order to provide appropriate management and improved quality of life for the community at large.

It is said that the Post COVID-19 world will witness significant lifestyle changes. To qualify the healthcare professional and patient equation, interaction, approach and treatment models, whether in the physical or digital, tele-practice and tele-rehabilitation environment will undergo substantial changes. The threat of contracting COVID-19 calls for a review of rehabilitation care and patient’s interaction with clinicians on priority. Tele-practice may be resorted to on fast track which may ease the daily and re-occurring load on formal clinical care and prevent transmission of the disease to the patient and the health professional.

### Author`s Contribution:

**NM:** Conceptualization of work, designing of research, Analysis & Interpretation.

**GS:** Writing of Manuscript, Methodology, Literature Review, Finalization for publication and Responsible and Accountable for the accuracy or integrity of the work

**NM:** Critical revision of article.
